# Melatonin’s Impact on Wound Healing

**DOI:** 10.3390/antiox13101197

**Published:** 2024-10-02

**Authors:** Eun-Hwa Sohn, Su-Nam Kim, Sung-Ryul Lee

**Affiliations:** 1 Department of Bio-Health Convergence, Kangwon National University, Chuncheon 24341, Republic of Korea; ehson@kangwon.ac.kr; 2 Natural Products Research Institute, Korea Institute of Science and Technology, Gangneung 25451, Republic of Korea; 3 Department of Convergence Biomedical Science, Cardiovascular and Metabolic Disease Center, College of Medicine, Inje University, Busan 47392, Republic of Korea

**Keywords:** antioxidant, inflammation, injury, melatonin, oxidative stress, pineal gland, wound healing

## Abstract

Melatonin (5-methoxy-N-acetyltryptamine) is an indoleamine compound that plays a critical role in the regulation of circadian rhythms. While melatonin is primarily synthesized from the amino acid tryptophan in the pineal gland of the brain, it can also be produced locally in various tissues, such as the skin and intestines. Melatonin’s effects in target tissues can be mediated through receptor-dependent mechanisms. Additionally, melatonin exerts various actions via receptor-independent pathways. In biological systems, melatonin and its endogenous metabolites often produce similar effects. While injuries are common in daily life, promoting optimal wound healing is essential for patient well-being and healthcare outcomes. Beyond regulating circadian rhythms as a neuroendocrine hormone, melatonin may enhance wound healing through (1) potent antioxidant properties, (2) anti-inflammatory actions, (3) infection control, (4) regulation of vascular reactivity and angiogenesis, (5) analgesic (pain-relieving) effects, and (6) anti-pruritic (anti-itch) effects. This review aims to provide a comprehensive overview of scientific studies that demonstrate melatonin’s potential roles in supporting effective wound healing.

## 1. Introduction

**Figure 1:**
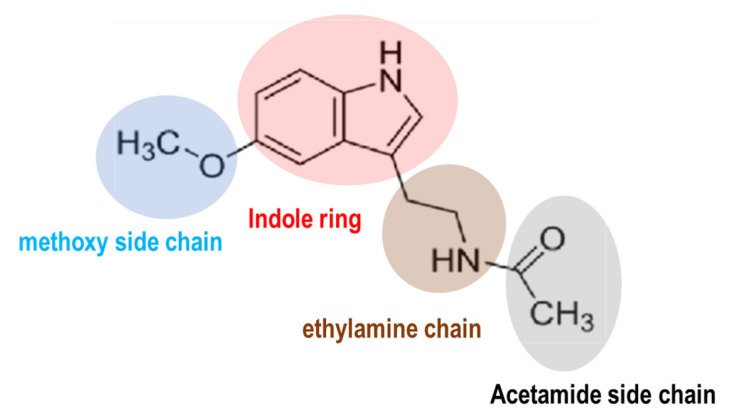
The chemical structure of melatonin. Melatonin structure is composed of an indole ring with an acetamide side chain (−CH_2_CONH_2_) and a methoxy side chain (−OCH_3_) on a benzene ring.

Melatonin is a molecule conserved chemically across prokaryotes to humans. The molecular structure of melatonin consists of an indole ring with an acetamide side chain (−CH_2_CONH_2_) and a methoxy group (−OCH_3_) attached to the benzene ring (Figure 1). The circadian clock orchestrates the synchronization of sleep–wake cycles, metabolism, and physical activity [1,2,3]. In humans, the central circadian clock resides in the suprachiasmatic nucleus (SCN) of the hypothalamus. Melatonin serves as a critical signaling molecule in the alignment of circadian rhythms [2,4,5]. Its synthesis and secretion are governed by light and darkness perception [4]. Several distinct functions of melatonin within the circadian system have been characterized [6,7]. Firstly, melatonin (5-methoxy-N-acetyltryptamine) is recognized as a neuroendocrine hormone naturally produced by the pineal gland, located centrally in the brain, posterior to the third ventricle. Currently, melatonin is recognized as a ubiquitous molecule found in all animal species [8,9,10], and substantial amounts have also been detected in higher plants, including fruits, seeds, and leaves [11,12,13]. The local production of melatonin is possible due to the widespread presence of melatonin-synthesizing enzymes in various tissues including the gastrointestinal tract [14], retina [15], and skin [16]. The widespread production of melatonin in various tissues may facilitate a rapid response to environmental cues and oxidative stress [17,18,19,20]. For example, immune cells synthesize substantial amounts of melatonin and express melatonin receptors, indicating a potential intracrine and paracrine role for melatonin in modulating immune responses [20,21]. Among extrapineal organs, only the retina houses the circadian pacemaker responsible for driving rhythmic melatonin synthesis [22]. The classification of melatonin as a neurohormone has been challenging due to its nonclassical properties as a hormone and its similar mode of action to vitamin D and vitamin A [8]. In addition to its role in regulating sleep, melatonin exhibits antioxidant properties and plays a role in immune function [23,24] and the regulation of various hormones, including estrogen, testosterone, cortisol, and insulin, among others [18,25]. Currently, the use of melatonin as an adjunct therapy is supported for a variety of conditions, including (1) macular degeneration, (2) glaucoma, (3) protection of the gastric mucosa, (4) management of irritable bowel syndrome, (5) treatment of arterial hypertension, (6) diabetes, (7) alleviation of chemotherapy and radiation side effects in cancer patients, (8) management of hemodialysis in renal insufficiency, and, notably, (9) circadian rhythm sleep disorders such as jet lag, delayed sleep phase syndrome, and age-related sleep disturbances [26,27,28,29,30,31,32,33,34].

An injury is defined as “physical damage that occurs when the human body is abruptly exposed to energy levels that surpass the threshold of physiological tolerance, or as a result of the deficiency of one or more essential elements, such as oxygen” [35]. Injuries that result in wounds, which are often unavoidable in daily life, can be classified based on their medicolegal significance—such as being intentional, accidental, or of undetermined intent—or according to their modality, including traumatic injury, thermal injury, falls, drowning, poisoning, and injuries caused by animals. The body’s physiological response to any wound involves the inflammation, proliferation, and remodeling phases, and those processes overlap each other both temporally and spatially, within the wound site [36,37,38]. Infection, poor blood supply (ischemia), chronic inflammation, and systemic diseases such as diabetes and autoimmune disorders, as well as malnutrition, can compromise the body’s ability to heal wounds [35,39]. As individuals age, they often experience a decline in immune system function, resulting in increased susceptibility to infections and slower wound healing in the elderly. Moreover, common underlying health conditions in older adults can impair wound healing by reducing blood flow to the affected area, thereby hindering the delivery of essential oxygen and nutrients required for tissue repair. The cost of wound management is largely overlooked, and its impact is often underestimated [40]. Inadequate wound healing can lead to functional limitations, particularly when the wound affects joints, muscles, or other essential structures.

In theory, melatonin might contribute to promoting wound healing through its ability to diminish inflammation, shield cells from oxidative-stress-induced damage, and regulate immune responses crucial to the healing mechanism [18,41,42]. Studies have shown that melatonin can accelerate wound closure, improve tissue regeneration, and enhance the overall healing of various types of wounds, including cuts, burns, and ulcers [18]. The investigation of melatonin’s wound healing properties, due to its potential to enhance the body’s natural healing processes, has garnered significant interest for its clinical applications [18]. In this review, we offer a narrative overview of scientific studies that elucidate the various roles of melatonin in promoting optimal wound healing.

## 2. Melatonin

### 2.1. Chemical Properties of Melatonin and Its Metabolites

Indole serves as a core structure in various natural compounds, including the melatonin precursor tryptophan, serotonin, tryptamine, indole-3-acetic acid, and melatonin itself [43]. The indole ring is highly reactive toward electrophilic aromatic substitution reactions, especially at the C-3 position of the pyrrole ring. Melatonin exhibits amphiphilic characteristics, displaying both hydrophilic (water-attracting) and lipophilic (fat-attracting) behaviors. Melatonin encounters no morpho-physiological barriers. It readily traverses the blood–brain barrier [44], the placenta [45], and other potential cellular barriers [46]. Its relative hydrophobicity allows the indole ring to interact with lipid membranes and hydrophobic pockets in proteins. This characteristic is essential for its role in biological systems, which includes membrane crossing and protein binding. The methoxy group in melatonin is characterized by their electron-donating properties, ability to stabilize conjugate bases, and influence on the chemical reactivity and physical properties of molecules. The acetamide group in the structure of melatonin contributes to its overall polarity, enhancing its solubility in aqueous environments. Additionally, the acetamide side chain can participate in hydrogen bonding due to its carbonyl (C=O) and amine (NH_2_) groups. This ability to form hydrogen bonds is crucial for melatonin’s interaction with its biological receptors, particularly melatonin receptors 1 (MT1R) and 2 (MT2R).

### 2.2. Biosynthesis of Melatonin

**Figure 2:**
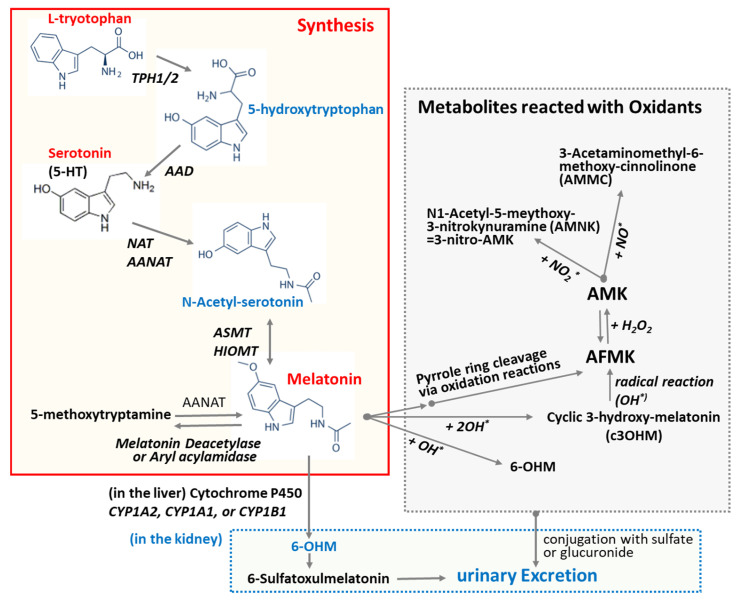
Biosynthesis of melatonin and its degrading pathway. L-Tryptophan and serotonin are precursors of melatonin. Melatonin synthesis from L-tryptophan sequentially involves four enzyme-catalyzed reactions: hydroxylation, decarboxylation, acetylation, and methylation. The oxidation of melatonin can lead to the formation of various oxidized forms and metabolites. Melatonin undergoes secondary metabolism in the kidney and is excreted in the urine as an inactive metabolite 6-OHM. Abbreviations: AAD: aromatic amino acid decarboxylase; AANAT: Aralkylamine N-acetyltransferase; ASMT: Acetylserotonin O-methyltransferase; AFMK: N1-Acetyl-N2-formyl-5-methoxykynuramine; AMK: N1-Acetyl-5-methoxykynuramine; HIMOT: Hydroxy-O-methyltransferase; TPH1/2: Tryptophan Hydroxylase 1/2; NAT: N-Acetyltransferase; 5-HT: 5-hydroxytryptamine; 6-OHM: 6-Hydroxymelatonin; OH*: hydroxy radical.

L-Tryptophan and serotonin are precursors of melatonin [47]. Unlike other endocrine organs, the pineal gland does not store melatonin for later release. Upon synthesis, melatonin is immediately released into the cerebrospinal fluid (CSF) and bloodstream [48]. This behavior of melatonin contrasts with that of many hormones, which are typically stored and released in response to specific stimuli. As depicted in Figure 2, melatonin synthesis from L-tryptophan involves a series of four enzyme-catalyzed reactions. These steps include hydroxylation by tryptophan hydroxylase (TPH) [49], decarboxylation by aromatic L-amino acid decarboxylase (AAD) [49], acetylation by N-acetyltransferase (SNAT) and/or arylalkylamine N-acetyltransferase (AANAT), and, finally, methylation by hydroxyindole-O-methyltransferase (HIOMT), also known as acetylserotonin O-methyltransferase. Melatonin production is linked to the activity of arylalkylamine N-acetyltransferase (AANAT), which is regulated by the SCN through nocturnal sympathetic release of norepinephrine [48]. Mammalian skin has the capacity to convert serotonin into melatonin through both AANAT-dependent and AANAT-independent mechanisms [49]. In addition to its endogenous production, melatonin can be readily acquired through dietary sources as it is also synthesized in various plants [50].

### 2.3. Melatonin Levels and Its Bioavailability

Nocturnal melatonin levels in blood are typically 10 to 20 times higher than daytime levels [51,52]. It is noteworthy that melatonin levels are roughly five times higher in children compared to adults [51,52]. Circulating melatonin can achieve concentrations of up to approximately 1 nM in blood. However, extrapineal melatonin levels vary by tissue type and can occasionally attain micromolar concentrations. Notably, melatonin levels in the gastrointestinal tract (GIT) are 10–100 times higher than those in serum, and the total melatonin content in the GIT has been estimated to be up to 400 times greater than in the pineal gland. This elevated GIT melatonin may originate from various sources, including adjacent tissues, bile, intestinal microbiota, or dietary intake [53]. However, the mechanisms governing melatonin production and regulation in extrapineal tissues remain poorly understood, as well as the impact of melatonin from these tissues on systemic levels in the circulatory system.

Melatonin has a short half-life, ranging from 30 to 50 min depending on the species [54]. In the bloodstream, approximately 70% of melatonin is bound to albumin, while the remaining 30% distributes into the surrounding tissues. The bioavailability of orally consumed melatonin is approximately 18.9% with an obvious sexual difference [55,56]. Orally administered melatonin rapidly appears in the bloodstream and is subsequently eliminated or metabolized within approximately 19 h [57,58]. This rapid elimination may be attributed to a relatively high first-pass metabolism of approximately 30% in the liver. It has been recommended that the maximum dose of melatonin should not exceed 3 mg/kg, equivalent to about 35 mg for a 75 kg adult. This dosage is necessary to accurately assess the effects of melatonin mediated by melatonin receptors at physiological levels. Furthermore, melatonin effects observed at concentrations below 0.1 μmol/L are deemed physiologically relevant, whereas effects at higher concentrations are considered pharmacologically significant [59].

The skin may be an optimal site for both local treatment through topical application and systemic therapy via transdermal delivery, potentially maintaining stable plasma melatonin levels through continuous release from the stratum corneum [60,61]. In contrast to oral administration, transdermal delivery of melatonin leads to a substantial delay in achieving peak blood levels, supporting the hypothesis that melatonin may accumulate in the skin [62]. When comparing the efficiency of transdermal delivery, alcohol-based melatonin solution enhances its transdermal delivery compared to a cream-based formulation [62]. However, further research is required to fully determine the efficacy of melatonin delivered through various dermatological applications in achieving systemic effects.

### 2.4. Melatonin Metabolism

Melatonin metabolism primarily occurs in the liver, where it undergoes hydroxylation mediated by cytochrome P450 monooxygenase enzymes, particularly CYP1A2. Melatonin metabolites are excreted after conjugation with either sulfuric or glucuronic acid in the kidneys. However, melatonin metabolism can vary depending on the specific tissues involved [54]. In the central nervous system (CNS), the oxidative cleavage of the pyrrole ring predominates, and 6-OHM is not detected following melatonin injection into the cisterna magna. This observation suggests that, within the pineal gland, a greater proportion of melatonin may be released into the CSF via the pineal recess than into the systemic circulation.

It has been predicted that the multiple physiological functions of melatonin may be mediated and/or further amplified by its metabolites, as melatonin metabolism produces numerous structurally diverse derivatives [63]. Oxidation of melatonin can lead to the formation of various oxidized derivatives and metabolites (Figure 2). The commonly studied oxidized forms of melatonin include N1-acetyl-N2-formyl-5-methoxykynuramine (AFMK), N1-acetyl-5-methoxykynuramine (AMK), and 6-hydroxymelatonin (6-OHM). The primary cleavage product of melatonin is AFMK, which is further metabolized into AMK. Both AFMK and AMK are generated via the kynuric acid pathway [63]. As depicted in Figure 2, melatonin can be converted nonenzymatically to AFMK through the action of reactive oxygen species (ROS) or ultraviolet B (UVB) light [64]. This process might include the generation of 3-hydroxymelatonin, 2-hydroxymelatonin, melatonin 2-indolinone, 3-hydroxymelatonin 2-indolinone, and melatonin dioxetane as intermediate products. Alternatively, melatonin can be demethylated to N-acetylserotonin by the enzymes CYP2C19 or CYP1A2 [49]. In the indolic degradation pathway, melatonin deacetylase converts melatonin to 5-methoxytryptamine, which is subsequently oxidized by monoamine oxidase to 5-methoxyindoleacetaldehyde. This intermediate can then be further metabolized to 5-methoxyindoleacetic acid by aldehyde dehydrogenase or to 5-methoxytryptophol by alcohol dehydrogenase. The cyclic 3-hydroxymelatonin (c3-OHM) is the product of the reaction of melatonin with hydroxy radical (HO^*^) and also possesses antioxidant properties [65]. The presence and concentration of c3-OHM may reflect increased oxidative stress and suggest an upregulation of melatonin’s protective mechanisms [65]. N1-Acetyl-N2-hydroxy-5-methoxytryptamine (AHMT) is a minor metabolite formed through the hydroxylation of melatonin at the N_2_ position. Melatonin undergoes secondary metabolism in the kidneys, where it is converted into an inactive metabolite, 6-OHM, and subsequently excreted in the urine. 6-OHM is more hydrophilic than AFMK [55].

### 2.5. Key Proteins That Interact with Melatonin

**Figure 3:**
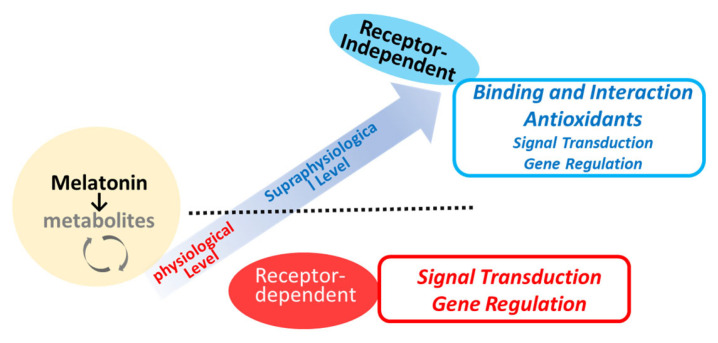
Potential mechanisms of melatonin action in relation to local concentrations.

Melatonin receptors (MTRs) are part of the 7-transmembrane G-protein-coupled receptor (GPCR) family, specifically, within the rhodopsin family (Class A) [66,67]. These receptors are activated by endogenous ligands such as melatonin and N-acetylserotonin, as well as by therapeutic agents developed for clinical use, including ramelteon, agomelatine, and tasimelteon [4,68,69]. As briefly depicted in Figure 3, melatonin has both receptor-dependent and receptor-independent actions [6,7,70]. There are two main types of MTR (MT1R and MT2R) with 60% homology at the amino acid level identified so far [34,59,66]. MT1R is primarily linked to facilitating the phase-shifting effects of melatonin on the circadian rhythm [71,72]. Additionally, it plays a role in neuroprotective effects [71] and the modulation of blood pressure. The MT2R is extensively distributed in the brain, gastrointestinal tract, and various peripheral tissues. The complex interactions of melatonin with its receptors contribute to its versatility in influencing circadian rhythms, sleep regulation, and other biological functions [18]. The subtle differences in pharmacological and signaling properties between MT1R and MT2R may be critical in understanding the specific physiological effects of melatonin [73]. The desensitization of MT1R or MT2R to melatonin may only occur after prolonged exposure to melatonin (>5 h) [73]. MTR can be regulated in a homologous manner, that is, by melatonin itself, via G-protein uncoupling, phosphorylation, internalization, and down-regulation of MT1R/MT2R [73]. Other stimuli such as the photoperiod [74] or estradiol [75] may also participate in the regulation of MTRs. MT1R and MT2R are regulated differently by physiological melatonin concentrations (30–400 pM) compared to supraphysiological concentrations (1–1000 nM) [64]. The melatonin concentration that promotes gene expression corresponds to the physiological peak of plasma melatonin observed at night. In contrast, higher melatonin concentrations do not have a similar effect on gene expression [64]. A significant limitation in studies investigating melatonin receptor signaling is the difficulty in identifying a physiological stimulus that triggers melatonin-receptor-mediated intracellular signaling pathways [68]. The melatonin metabolite 6-OHM acts as a full agonist of the MT1R and MT2R. Recent experimental findings have identified that melatonin and its metabolites (e.g., 6-OHM and AFMK) can act as ligands for the aryl hydrocarbon receptor (AhR), a ligand-activated transcription factor that responds to environmental and endogenous signals [76], as well as for the peroxisome proliferator-activated receptor (PPAR) at high concentrations [77]. This discovery suggests a potential mechanism by which melatonin and its metabolites, when present at high local concentrations, may help mitigate cellular damage induced by oxidative stress. Further investigation is needed into the direct effects of melatonin, including actions that occur through non-receptor-mediated pathways, in various stages of wound healing, and beyond the specific functions of MT1R and MT2R.

The small lipophilic structure of melatonin suggests the possibility of intracellular binding sites and functions as well. NQO2 (NAD(P)H:quinone oxidoreductase 2; E.C. 1.6.99.2) [78] and retinoid-related orphan receptor alpha (RORα) have been proposed as intriguing proteins that interact with melatonin as melatonin-interacting proteins [70]. It is worth noting that many of the actions of melatonin are mediated through receptor-independent activities and non-genomic actions, meaning effects that do not involve changes in gene expression. Melatonin also acts as a potent antioxidant [42,63,65,79,80,81], and some of its effects may be mediated through direct interaction with reactive oxygen species and other signaling pathways. As an epigenetic action [82], which modifies DNA and histones, melatonin has the potential to boost mRNA expression for various histone deacetylase (HDAC) isoforms in the C17.2 neural stem cell line [83]. Importantly, a significant elevation in histone H3 acetylation, a process intricately associated with chromatin remodeling and gene transcription, has been observed following 24 h of melatonin treatment [83].

Melatonin can participate in signaling pathways through direct protein interactions, independent of its interactions with melatonin receptors (Figure 3). For example, melatonin has been shown to antagonize calcium-regulatory protein calmodulin (CaM) [84,85,86,87]. Melatonin and its analogs, such as 6-chloromelatonin, 6-hydroxymelatonin, and luzindole, bind to calmodulin in a reversible and calcium-dependent manner [88]. From a functional perspective, direct interaction between melatonin and calcium-saturated CaM inhibits CaM-dependent enzymes such as cAMP phosphodiesterase, nitric oxide synthase, and CaM-kinase II as a functional consequence of this interaction [84,86,87]. Melatonin also has the potential to regulate cytoskeletal organization [89,90]. It is suggested that, at low concentrations (~pM), melatonin’s effects on the cytoskeleton are mediated by its antagonism of Ca^2+^/CaM. At higher concentrations (μM), melatonin nonspecifically binds to tubulin, which overrides its antagonism of Ca^2+^/CaM [91]. These findings support the hypothesis that, under physiological conditions, melatonin synchronizes various body rhythms through cytoskeletal rearrangements mediated by its calmodulin antagonism.

## 3. Roles of Melatonin for Supporting Wound Healing

**Figure 4:**
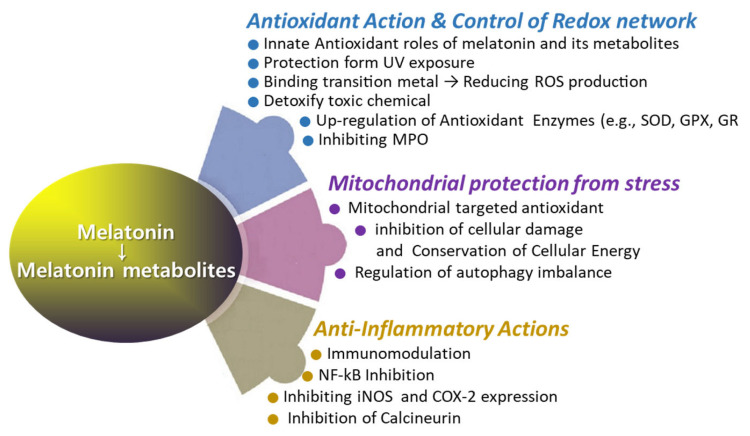
The roles of melatonin in antioxidant defense, anti-inflammatory response, and mitochondrial protection.

**Figure 5:**
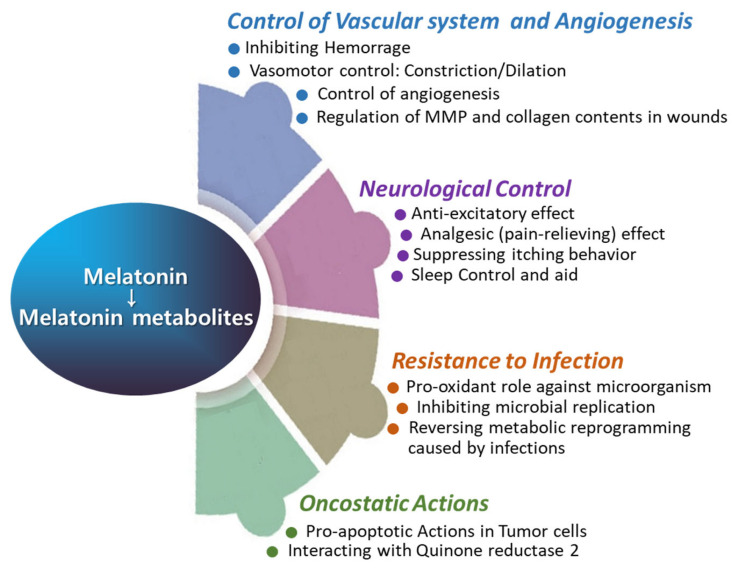
The roles of melatonin in vascular function, angiogenesis, neurological regulation, infection control, and oncostatic activity.

**Figure 6:**
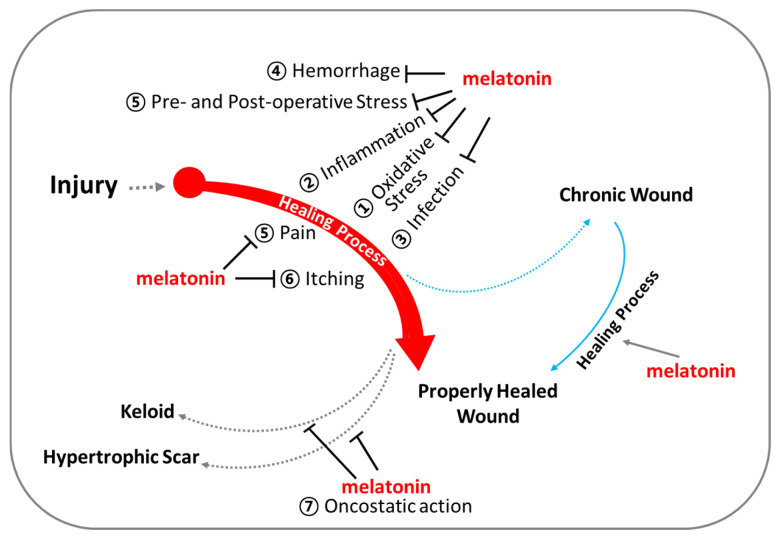
Melatonin for optimal support of wound healing. Beyond regulating circadian rhythm, melatonin exhibits (1) powerful antioxidant capacities, (2) anti-inflammatory actions, (3) infection control, (4) regulation of vascular reactivity and angiogenesis, (5) analgesic (pain-relieving) effects, (6) anti-itch (anti-pruritic) effects, and (7) oncostatic effects.

The use of melatonin is gradually increasing; however, reports on its effects on wound healing are occasionally inconsistent [92,93,94,95]. Several studies have demonstrated that melatonin treatment significantly enhances collagen synthesis and promotes accelerated wound healing [92,93]. However, there are reports that exogenous melatonin reduces collagen synthesis and epithelial proliferation, thereby impairing wound healing in both normal and pinealectomized rats [94]. Numerous reports have proposed melatonin as a potential aid in wound healing [18,96,97,98,99,100,101,102]. Notably, melatonin has been investigated for its role in regenerating the morphological and functional capacity of injured muscle after crush injuries [103] and improving recovery from muscle fatigue following intensive exertion [18]. Additionally, melatonin has shown promise in enhancing bone formation, reducing osteoporosis, and improving the success of bone grafting [104]. The rationale behind using melatonin in treating wound healing lies in its multifaceted physiological effects that can positively influence the various stages of the wound healing process (Figure 4 and Figure 5). Melatonin’s roles span enhancing wound healing, reducing scar formation, protecting against ischemia–reperfusion injury, reducing inflammation and pain, modulating immune responses, offering photoprotection, and mitigating oxidative stress. Melatonin may promote faster healing by enhancing the viability and function of cells involved in tissue repair, owing to its potent antioxidant properties. Additionally, melatonin may promote faster healing via stimulation of collagen synthesis in fibroblasts. The melatonin’s beneficial role in enhancing blood flow to the wound area can support faster wound healing via improving tissue oxygenation and nutrients (Figure 5 and Figure 6). Melatonin may mitigate excessive inflammation and reduce the risk of complications such as infection, thereby supporting optimal wound healing outcomes (Figure 5). Additionally, the oncostatic effects of melatonin may be beneficial in preventing the formation of hypertrophic scars and keloids [105]. Numerous trials are focused on developing melatonin–polymer wound healing patches with improved mechanical properties to better support and enhance the healing process [98,99,106,107].

### 3.1. Antioxidant Roles in the Wound Healing

Melatonin directly detoxifies various reactive oxygen species (Figure 2), such as hydrogen peroxide (H_2_O_2_), hydroxyl radical (OH^*^), peroxyl radicals (ROO−), and singlet oxygen (^1^O_2_) [63,65,80,81,108]. Additionally, melatonin neutralizes reactive nitrogen species (RNS), including nitric oxide radicals (NO) and peroxynitrite (ONOO−) [109]. Among them, melatonin is particularly effective in neutralizing hydroxyl radicals, which are considered the most damaging of all free radicals due to their ability to react with nearly all organic molecules [108]. Recent studies suggest that melatonin is a potent antioxidant [18,65,79,110,111,112]. Melatonin’s interaction with ROS and RNS is a prolonged process involving many of its derivatives, referred to as the free radical scavenging cascade [113]. This cascade reaction represents a unique property of melatonin, distinguishing it from conventional antioxidants. Melatonin’s potent antioxidant activity is attributed to its distinct structural characteristics, as well as those of its biologically active metabolites, which possess similar molecular structures [63]. AFMK and AMK are major metabolites produced from the oxidation of melatonin via the kynuric pathway, and they are believed to contribute to melatonin’s overall antioxidant effects [63]. Another melatonin metabolite, 6-OHM, scavenges more potently free radicals and reduces more oxidative stress in this regard than melatonin itself. As previously mentioned, the quantity of c3-OHM produced is associated with in vivo hydroxyl radical generation [65].

Melatonin also mitigates oxidative stress through its involvement in a metal-chelating cascade [114,115]. 6-OHM offers protection against neurotoxicity induced by iron (Fe^2+^). Additionally, melatonin and its metabolites (e.g., AFMK and c3-OHM) are all capable of fully inhibiting oxidative stress induced by Cu(II)–ascorbate mixtures via metal chelation [114]. These findings suggest that melatonin may potentially aid in metal detoxification and further decrease the generation of free radicals [116]. Unlike melatonin, melatonin receptor agonists may have less antioxidant capacity. For example, agomelatine failed to show direct antioxidant activity when evaluated for iron chelation, reduction of synthetic DPPH (2,2-Diphenyl-1-picrylhydrazyl), and scavenging of ROS [117].

The ineffectiveness of conventional antioxidants in reducing the severity of ROS-related diseases may be due to their inability to accumulate within mitochondria, where free radical generation is most intense. Melatonin appears to be highly specific as a free radical scavenger due to its significant lipophilicity, which allows it to readily access intracellular molecules (Figure 4). When melatonin crosses cellular membranes, it primarily accumulates near the polar head groups of membrane phospholipids within the lipid bilayer. This localization enables melatonin to function effectively as a free radical scavenger and may contribute to the protection of membranes from oxidative damage [118]. Through a similar mechanism, melatonin can stabilize the inner mitochondrial membrane, potentially enhancing the activity of the electron transport chain [119]. This action of melatonin within the mitochondria may provide an additional indirect mechanism by which it mitigates molecular damage to essential molecules caused by ROS [120]. Melatonin is synthesized in the mitochondria of nearly all cells [110] and acts as an antioxidant, analogous to mitochondria-targeted antioxidants such as MitoE and MitoQ. In a rat model of acute sepsis induced by lipopolysaccharide and peptidoglycan, which is marked by severe inflammation, mitochondrial dysfunction, and early organ damage, melatonin exhibits efficacy comparable to, and in some cases superior to, that of MitoE and MitoQ [121]. Melatonin may be the most readily available agent for mitigating molecular damage and reducing mortality in septic patients. Its efficacy in scavenging hydroxyl and other free radicals, as well as its indirect antioxidant properties, has been consistently documented in numerous independent studies [121]. During radiotherapy, approximately 95% of patients experience radiation-induced skin injury, which can range in severity from mild erythema to more severe conditions such as moist desquamation and ulceration [122]. Melatonin may be effectively utilized for both the prevention and treatment of radiation-induced skin injury [31,123,124]. Furthermore, animals exposed to whole-body irradiation and treated with melatonin have shown increased survival rates [125].

Alongside its direct role as a free radical scavenger, melatonin’s indirect antioxidant effects may be mediated through its interaction with antioxidant enzymes to regulate their activity and/or by modulating the gene expression of these enzymes [126,127]. Antioxidant enzymes, such as glutathione peroxidase (GPX) [126] and superoxide dismutase (SOD) [127], display endogenous rhythms under normal light–dark cycles, with their activities correlating with melatonin levels. Melatonin can modulate the expression levels of antioxidative enzymes through several mechanisms. Melatonin can modulate immediate early gene (IEG) transcription by inhibiting protein kinase A (PKA) and cAMP response element-binding protein/activating transcription factor (CREB-ATF), thereby influencing gene transcription regulation and the concentration of antioxidant enzymes [73]. Melatonin can also activate the Nrf2–antioxidant responsive element (ARE) pathway [128], which regulates antioxidant and detoxification genes such as heme oxygenase-1 (HO-1), glutathione-s-transferase (GST), catalase (CAT), superoxide dismutase (SOD), and NADPH quinone dehydrogenase 1 (NQO1) [129,130]. Differently, melatonin has the capability to directly interact with myeloperoxidase (MPO; EC 1.11.1.7), thereby influencing the formation of MPO intermediates and their decay rates [131]. Collectively, melatonin serves as a powerful antioxidant via the free radical scavenging cascade [113] and stimulates antioxidant defense via regulating gene expression of antioxidant enzymes.

Although oxidants serve as cellular messengers to promote healing [132], excessive levels of ROS can trigger epigenetic modifications, disrupt normal cell signaling, promote uncontrolled cell proliferation, initiate cellular damage, and stimulate inflammatory processes [133]. Antioxidants at the injury site may be rapidly inactivated and depleted. Consequently, it has been proposed that administering antioxidants and enzymes with direct antioxidant properties could accelerate wound healing and prevent the formation of chronic wounds [134,135]. Additionally, antioxidants may help minimize scar formation by promoting a more controlled and organized deposition of collagen. As determined in a rat model [136], melatonin is an important antioxidant that can be used alone or in combination with N-acetylcysteine to increase McFarlane flap viability and prevent distal necrosis. Oral administration of melatonin (20 mg/kg/day) during fat grafting in mouse scalps resulted in better adipose tissue integrity, reduced inflammation, smaller oil cysts, and a lower degree of fibrosis [137]. Additionally, melatonin and NAC have been suggested as potential treatments for chronic skin ulcers due to their ability to promote angiogenesis by mitigating excessive free radical formation, which otherwise inhibits the effects of vascular growth factors [136]. In the context of palatal wound healing, tissue treated with melatonin exhibited significant clinical improvement within the first week post-surgery, indicating an accelerated rate of healing [138].

### 3.2. Anti-Inflammatory Roles in Wound Healing

Many studies have identified melatonin as a positive regulator of the immune system [64,139,140]. Pinealectomy and other experimental methods that impair melatonin synthesis and secretion induce immunodepression, a condition that can be alleviated by melatonin administration. Melatonin appears to possess an immunoenhancing effect, which becomes especially prominent in immunodepressed conditions [140]. Melatonin has also been shown to restore cellular Zn^2+^ levels in the thymus of old animals [141]. Melatonin enhances both innate immunity (e.g., phagocytosis) and cellular immunity (e.g., antigen presentation). Melatonin stimulates the production of progenitor cells for granulocytes, macrophages, and natural killer (NK) cells. It also enhances the production of interleukins IL-2, IL-6, and IL-12, and increases T-helper cell levels, particularly CD4+ cells, while decreasing CD8+ cells [142]. Melatonin is considered a potential therapeutic agent for enhancing immune function in the elderly and individuals with compromised immune systems [139,143]. The regulation of immune function by melatonin likely involves cAMP signal transduction [144], L-type Ca^2+^ channels, and glutathione [139,143].

Melatonin may act as an anti-inflammatory agent by inhibiting certain immune responses [139,145]. In the progression of distant organ injury (DOI) following severe burns, the initial inflammatory response can be extensive and become uncontrolled, leading to increased inflammation that impedes healing and induces a generalized catabolic state, thereby delaying the recovery process [146]. According to a systematic review on burn-wound-induced DOI, melatonin has emerged as a promising therapeutic candidate for short-term burn management [100]. Melatonin treatment significantly reduced lipid peroxidation and levels of pro-inflammatory molecules, such as tumor necrosis factor (TNF)-α, C-reactive protein, and MPO, in burn-induced DOI. Additionally, melatonin treatment notably increased the levels of anti-inflammatory molecules, including IL-10, in the affected tissues.

In various preclinical models, melatonin has been demonstrated to be a potent inhibitor of high mobility group box 1 (HMGB1) activation, which is triggered by inflammation and oxidative stress [147]. HMGB1 is a prototypic damage-associated molecular pattern (DAMP) molecule and is implicated in several inflammatory disorders [148]. Additionally, melatonin has been demonstrated to inhibit the activation of Nuclear Factor-kappa B (NF-κB) by suppressing the phosphorylation of IκBα and preventing the nuclear translocation of the p65 subunit during inflammation and immune responses [129,149,150,151]. Indeed, there are no reports indicating that melatonin directly interacts with NF-κB [34,70]. Instead, melatonin inhibits NF-κB activity through its antioxidant properties, thereby regulating the expression of genes involved in inflammation. It promotes the production of anti-inflammatory cytokines, such as IL-10, while inhibiting pro-inflammatory cytokines, such as TNF-α. Additionally, in T lymphocytes, melatonin stimulates the production of IL-2 and IL-4 [140].

Melatonin and its metabolites, AFMK and AMK, selectively inhibit the gene expression of the pro-inflammatory enzyme COX-2 (cyclooxygenase-2) induced by LPS (lipopolysaccharides) [152]. In contrast, the structurally related compound 6-methoxy-melatonin only partially attenuates the increase in COX-2 protein levels induced by the toxin. Similarly, melatonin prevents iNOS (inducible nitric oxide synthase) activation and reduces the levels of products from both COX-2 and nitric oxide. However, the endogenous antioxidant N-acetyl-cysteine (NAC) does not significantly reduce COX-2 levels [152]. 5-Lipoxygenase (5-LO) is another target of melatonin, contributing to its anti-inflammatory effects. 5-LO is a gene regulated by RORα, which is considered a potential target of melatonin and functions as a critical enzyme in the biosynthesis of leukotrienes. These leukotrienes play a dual role in the host defense system by acting as both mediators of inflammation and inflammatory agents themselves. Melatonin may repress 5-LO gene expression by binding to the nuclear receptor RORα in human B lymphocytes [153]. Additionally, 5-LO mRNA expression levels were elevated in the hippocampus of pinealectomized rats compared to sham-operated controls [154].

Melatonin may inhibit the production of cytokines involved in T-cell activation, which is mediated by calcineurin. As previously mentioned, melatonin interacts with calmodulin (CaM), potentially leading to the inhibition of calcineurin activity. This inhibition disrupts the activation of inflammatory responses, a mechanism similar to how calcineurin inhibitors are employed in the treatment of autoimmune diseases, such as atopic dermatitis [155]. The inhibitory effects of melatonin on calcineurin have been observed in neuroblastoma cells [156] and plants [85]. While melatonin’s potential to alleviate inflammatory responses through calcineurin inhibition is promising, further evidence is needed to clarify this role.

### 3.3. Infection Control in Wound Healing

Melatonin can influence pathogen infections in the host; however, pathogen infections can also impair the synthesis of melatonin [157,158,159,160]. It is noteworthy that the expression of melatonin-producing enzymes, such as AANAT and ASMT, can be influenced by pathogen infections or other disorders. These effects can arise from pathogen-mediated inflammatory responses, oxidative stress, altered neuroendocrine function, nutrient deficiencies, and direct interactions with the pathogen [157,158,159,160]. This alteration in melatonin secretion, either through disrupted rhythmicity or decreased levels, can contribute to bacterial translocation and an increased risk of sepsis. These effects can be reversed by melatonin administration.

Molecular mechanisms underlying the antimicrobial actions of melatonin have been proposed to involve (1) modulation of free radical formation to target specific pathogens [161]; (2) direct inhibition of bacterial replication; and (3) depletion of intracellular substrates, such as iron [18,158]. Melatonin has also demonstrated efficacy against a range of parasitic and viral infections, including Venezuelan equine encephalitis virus, viral hepatitis, viral myocarditis, and respiratory syncytial virus infections [18,158,160,162,163,164]. Additionally, melatonin delays the onset of disease caused by Semliki Forest virus inoculation and reduces mortality in mice infected with West Nile virus [165]. Malaria parasites use melatonin in their synchronization of development and this process can be abrogated with a melatonin receptor antagonist, luzindole [166]. Therapeutic effects of melatonin against viral infections have been suggested [167]. The pro-oxidant effects of melatonin have been found to be concentration and cell type dependent, without causing significant cytotoxicity [85,161]. Melatonin-induced ROS production appears to be mediated through melatonin’s interaction with calmodulin (CaM), as this effect is inhibited by chlorpromazine, a known calmodulin inhibitor [168]. However, it remains unclear whether melatonin’s pro-oxidant action during infection is similarly mediated through melatonin–CaM interactions. Melatonin stimulates the endogenous production of IFN-γ, IL-1β, and TNF-α, key cytokines involved in anti-viral action [165]. Viruses actively modify the metabolic pathways of host cells, including inducing mitochondrial dysfunction and Warburg-like metabolic reprogramming, to create an optimal environment for their replication and transmission [169]. Additionally, melatonin can reverse those reprogrammed metabolic pathways caused by influenza A virus infection, thereby alleviating the cellular energy crisis [170]. Melatonin’s antibacterial and anti-viral properties may promote faster, safer, and more efficient wound healing by preventing infections, thereby reducing the risk of complications and improving overall patient outcomes during the healing process.

### 3.4. Control of Vascular Reactivity in the Wound Healing

Vascular reactivity is essential for hemostasis as it promptly triggers vasoconstriction, platelet activation, and the coagulation cascade, all of which are vital for controlling bleeding and stabilizing the injury site. Additionally, it plays a key role in regulating the delivery of oxygen, nutrients, and immune cells to the injured area, which is crucial for effective wound healing. Melatonin may affect vascular reactivity [171]. It has been suggested as a potential first aid intervention in military settings where immediate medical care might be delayed [18]. In a murine model of traumatic colon injury (TCI), the prophylactic administration of melatonin effectively extended post-trauma survival by restoring gut homeostasis disrupted by TCI [172]. Postpartum hemorrhage, a serious condition associated with high morbidity and mortality in women after childbirth, whether vaginal or via cesarean section, may benefit from melatonin administration prior to surgery [173]. This approach has been shown to reduce blood loss, lower pain scores, and decrease the need for postoperative opioids. Additionally, studies have demonstrated that the administration of beta-hydroxybutyrate (BHB) and melatonin extends survival following severe hemorrhage, a frequent complication of traumatic injuries [174]. Melatonin modulates blood–brain barrier (BBB) endothelial functions by inhibiting matrix metalloproteinase-9 (MMP-9) [175]. It binds tightly to the active site of MMP-9, thereby reducing its catalytic activity [176].

Melatonin administration seems to lower sympathetic activity, leading to changes in heart rate [177]. However, variations in blood flow and vascular resistance across different organs are observed due to the presence of different melatonin receptor (MTR) subtypes and their complex mechanisms of action [178]. Melatonin activates two receptor subtypes in vascular smooth muscle: MT2R, which may promote relaxation, and another subtype that mediates vasoconstriction [179]. In a study of healthy men, melatonin administration significantly reduced blood pressure, the pulsatility index of the internal carotid artery, and catecholamine levels within 90 min [171]. Melatonin directly influences the contractile state of cerebral arteries by increasing vascular tone through the activation of melatonin receptors, which mediate the inhibition of large-conductance Ca^2+^-activated K+ (BKCa) channels [180]. In contrast, in pulmonary arteries, melatonin promotes vasodilation, contributing to resistance against hypoxia [178]. Both acute and chronic melatonin treatment induces vasodilation in pulmonary arteries, enhancing neuro-cardiopulmonary resonance. Furthermore, antecedent melatonin administration protects the kidneys from ischemia–reperfusion injury by enhancing autophagy [181].

Vascular reactivity and angiogenesis are interconnected through mechanisms involving endothelial cell function, growth factor regulation, and inflammatory responses, collectively governing blood vessel formation and function in both physiological and pathological contexts. Angiogenesis is critical for supporting fibroblast activity, collagen synthesis, and the formation of granulation tissue. Melatonin exerts context-dependent regulatory effects on angiogenesis. Depending on the specific physiological or pathological conditions, melatonin can either promote or inhibit neovascularization [95]. The use of injectable melatonin-loaded carboxymethyl chitosan (CMCS)-based hydrogel significantly enhanced wound closure rates, notably stimulated granulation tissue proliferation and re-epithelialization, and accelerated collagen deposition in a rat model of circular full-thickness cutaneous wounds [93]. Melatonin seems to be involved in the promotion of angiogenesis and VEGFR protein expression in the early stages of wound healing [95]. Additionally, it substantially reduced the expression of pro-inflammatory proteins, such as COX-2 and iNOS, in the later stages of wound healing [93]. In a rat model of dorsal skin incision injury, intramuscularly administered melatonin positively influenced angiogenesis during wound healing [182]. This effect was associated with increased vascularization, a higher density of collagen fibers, and elevated infiltration of macrophages and lymphocytes in the wounded area [182]. In a rat model of nasal septal perforation—a chronic mucosal wound—melatonin administration accelerated healing by enhancing epithelial regeneration, cartilage repair, capillary density, and granulation tissue formation [183]. Notably, the observed increase in capillary density and granulation tissue with melatonin treatment indicates improved angiogenesis. Consequently, melatonin may facilitate wound repair by promoting angiogenesis [95].

### 3.5. Neurologic and Analgesic (Pain-Relieving) Effect in the Wound Healing

Melatonin’s neurological roles in wound healing are associated with pain modulation, promotion of sleep and recovery, and regulation of stress response. Stress may disrupt inflammatory and immune responses, which can impair normal wound healing and potentially lead to latent infections [184,185]. Surgery is a significant physical stressor. Anesthesia and surgery have a significant impact on melatonin secretion due to several factors related to the physiological and psychological stress [186]. The body’s stress response includes the activation of the hypothalamic–pituitary–adrenal (HPA) axis, which can alter the secretion of various hormones, including melatonin. For example, elevated levels of cortisol, a stress hormone, can inhibit melatonin secretion. Melatonin mitigates attention deficit/hyperactivity disorder (ADHD) associated with psychological stress induced by atopic dermatitis [187]. In an NC/Nga atopic-like mouse model, melatonin supplementation could correct aberrant hypothalamic–pituitary–adrenal (HPA) axis responsiveness and over-reactivity of the sympathetic–adrenal–medullary system to psychosocial stress caused by atopic dermatitis [187]. This finding suggests that a cortisol–melatonin imbalance in atopic dermatitis might contribute to dopamine dysfunction by causing or enhancing neurodegeneration. This pathological event may be mitigated by supplementing the reduced levels of melatonin [187]. The mechanisms underlying melatonin’s observed anxiolytic effects are not fully understood. It has been proposed that melatonin’s anxiolytic properties may be linked to its ability to suppress the activation of the sympathetic nervous system, the renin–angiotensin–aldosterone system (RAAS), and the hypothalamic–pituitary–adrenal (HPA) axis [188]. Melatonin demonstrated efficacy and safety in treatment of nociceptive and neuropathic pain in several studies on animal models and clinical trials [189]. The beneficial impact of sublingual melatonin as an anxiolytic and sedative premedication agent has been identified in young females undergoing cesarean section under spinal anesthesia [190]. Melatonin premedication as an oral dose of 3 mg administered the night before surgery reduced incidence of postoperative delirium and cognitive dysfunction in various urological and gastrointestinal surgeries under general anesthesia [191]. Post-traumatic and post-surgical sensory disturbances are common complications associated with zygomaticomaxillary (ZMC) complex fractures that involve the infraorbital nerve. Prophylactic administration of melatonin has been shown to provide significant clinical benefits, including reduced postoperative pain and enhanced sensory recovery [192].

Melatonin’s analgesic properties have long been of great interest in alleviating pain associated with fibromyalgia, headaches, irritable bowel syndrome, chronic back pain, and rheumatoid arthritis [189]. The analgesic effects in wound healing are vital for ensuring patient comfort, promoting faster and more effective healing, preventing complications, and improving overall patient outcomes. Studies on both humans and animals have shown that pain also delays the healing of standardized punch biopsy wounds [193]. Melatonin may have analgesic (pain-relieving) effects [4,194,195] as well as affecting inflammatory [196] and chronic neuropathic pain [197]. The positive influence of melatonin as a preemptive analgesic for pain during periodontal flap surgery [196], lumbar laminectomy and discectomy [195], cesarean sections [194], and removal of impacted third molars [198] has been demonstrated. Melatonin demonstrated a preemptive analgesic effect comparable to that of the gold-standard analgesic ketorolac, while also exhibiting anti-inflammatory properties [196]. Melatonin can serve as an adjuvant drug for anesthesia during the perioperative period [186]. At the molecular level, our understanding of how melatonin functions as an anesthetic is still in its early stages. The possible mechanisms behind melatonin’s analgesic properties are thought to include the normalization of circadian rhythms and its inherent analgesic effects, which are mediated through receptors and various neurotransmitter systems including the GABA (γ-aminobutyric acid type A) receptor [199,200].

### 3.6. Anti-Itch (Pruritus) Effect of Melatonin

Itching, or pruritus, is often overlooked in clinical settings due to its subjective nature, the lack of standardized assessment tools, and a primary focus on more urgent or severe conditions. Different types of wounds and their respective stages of healing can induce varying degrees of itching. For instance, hypertrophic scars and burns can cause intense itching that may persist for years after the wound has healed, whereas venous ulcers typically produce only mild itching. Itching can contribute to delayed wound healing and recurrent infection by causing additional tissue damage, increasing the risk of infection, and perpetuating inflammation. Although research on the specific effects of melatonin on pruritus remains limited, emerging evidence suggests that it may offer potential benefits in the treatment of itchy skin [201]. In mouse models of acute and chronic pruritus, both systemic and local administration of melatonin significantly reduced acute itching induced by Compound 48/80 and chloroquine, as well as chronic itching induced by imiquimod and acetone–ether–water. These findings suggest that melatonin may alleviate both acute and chronic pruritus through its interaction with melatonin receptors, along with its antioxidant and anti-inflammatory effects [201]. Thymic stromal lymphopoietin (TSLP), a type I cytokine in the IL-2 family, is recognized as a key contributor to the pathogenesis of various allergic diseases [202]. TSLP expression can be triggered by environmental stimuli, including allergens, proteases, bacterial, viral, and parasitic infections, pattern recognition receptor ligands, and chemicals [202]. In human nasal epithelial cells [203] and an experimental atopic march mouse model [204], melatonin has been shown to reduce TSLP expression, which acts as a pruritus signal and exacerbates the progression of the atopic march. In clinical settings, two pilot randomized clinical trials demonstrated significant anti-pruritic effects in patients with chronic liver disease [205] and uremia [206] when treated with daily melatonin administration. Additionally, sleep disturbances in patients with atopic dermatitis (AD) have been linked to the severity of pruritus. Melatonin administration showed promising effects in reducing disease severity and improving sleep quality in children with AD [207]. Melatonin’s potential in alleviating pruritus is important for wound healing as it helps prevent re-injury, reduces the risk of infection, minimizes inflammation, preserves the integrity of the skin barrier, and enhances patient comfort and adherence to wound care protocols.

### 3.7. Oncostatic Role in the Wound Healing

Both hypertrophic scars and keloids are localized forms of fibrosis characterized by excessive collagen production in response to injury. Hypertrophic scars are typically confined to the original wound site, while keloids extend beyond the boundaries of the initial injury. Generalized fibrosis, in contrast, can affect multiple tissues and organs throughout the body. Shared underlying mechanisms in these conditions include fibroblast activation, dysregulation of collagen synthesis and degradation, and persistent inflammation. Hypertrophic scars typically improve over time, whereas keloids continue to evolve without entering a quiescent or regressive phase [208]. Intralesional corticosteroid injections have become a primary treatment for both hypertrophic scars and keloids [209,210]. These injections, used alone or in combination with other therapeutic modalities, help reduce fibroblast proliferation, collagen synthesis, and glycosaminoglycan production, while also suppressing pro-inflammatory mediators. Melatonin is preferentially taken up by, and possibly binds with, DNA in the nucleus, not unlike other lipid-soluble hormones [108]. It has clearly proven to be highly protective of DNA when exposed to oxidative radical attack [211], thereby helping to prevent cancer initiation [212]. Additionally, melatonin modulates angiogenesis with a dual effect; it promotes angiogenesis during wound healing while inhibiting it in pathological conditions such as cancer. Melatonin’s oncostatic potential, associated with its anti-inflammatory, antioxidant, and anti-fibrotic properties, has significant implications for the prevention and treatment of hypertrophic scars and keloids.

The effect of melatonin on hypertrophic scar and keloid formation in clinical settings remains uncertain due to the inherent variability in scar development, the complexity of the underlying pathogenesis, and limitations in measurement and predictive methods. However, in experimental settings, melatonin has shown potential in suppressing hypertrophic scars. For instance, intraperitoneal injection of melatonin in a rat model of burn wounds resulted in a reduction of the zone of stasis and decreased hypertrophic scar formation [101]. Additionally, in a rabbit ear model, melatonin administration effectively inhibited hypertrophic scar development [213]. This effect is likely related to the inhibition of the AKT/mTOR signaling pathway through direct interaction with PI3K, leading to decreased collagen and α-SMA production in fibroblasts associated with hypertrophic scars [213]. However, this effect was reversed by treatment with an AKT activator or the selective antagonist 4-PPDOT. Additionally, melatonin can inhibit fibroblast proliferation and promote apoptosis through its oncostatic effects, specifically targeting cell cycle regulation [105]. For instance, melatonin has been shown to suppress proliferation and induce apoptosis in fibroblasts isolated from human hypertrophic scars by modulating the expression of key regulatory proteins, including cyclin E, p53, and Fas (CD95) [214]. These proteins are integral to controlling cell growth, cell cycle progression, and apoptosis.

Keloid formation predominantly occurs in regions of the body with a high concentration of melanocytes. Keloids are characterized by an increased rate of collagen synthesis and a higher ratio of type I to type III collagen compared to normal skin and hypertrophic scars [215,216]. Additionally, keloid fibroblasts exhibit increased proliferation and decreased apoptosis relative to normal skin fibroblasts, likely due to disruptions in apoptotic mechanisms [208]. Given the malignant-like growth pattern of keloids, the anti-tumor chemotherapeutic agent 5-fluorouracil (5-FU) has been explored as a treatment for keloids [208]. Melatonin has been shown to significantly inhibit the survival of keloid fibroblasts (KFs) and to promote their apoptosis. It specifically targets KFs by inducing apoptosis, and inhibiting cell proliferation, migration, invasion, collagen contraction, and collagen synthesis [217]. Mechanistically, melatonin exerts its effects by inhibiting the cAMP/PKA/Erk and Smad signaling pathways through the MT2R on KFs. The combination of melatonin with 5-FU further reduces phosphorylation of Erk and Smad3, suggesting that this combination therapy could potentially offer a more effective treatment for keloids [217].

Fibrosis, in contrast to cutaneous wounds, frequently develops in soft tissues as a result of chronic inflammatory responses to injury or disease, resulting in disorganized and functionally impaired fibrotic tissue. Melatonin has demonstrated a capacity to mitigate liver damage in animal models of experimentally induced liver fibrosis, such as those involving carbon tetrachloride administration [218]. In these models, melatonin treatment inhibited hepatic stellate cell activation, reduced matrix metalloproteinase-9 (MMP-9) activity, and significantly decreased the expression of profibrogenic factors in a dose-dependent manner [218]. Melatonin has also been shown to decrease MMP-9 and MMP-2 activities in models of ethanol-induced gastric ulcers [219] and experimental colitis [220], with reduced tumor necrosis factor-alpha (TNF-α) expression. In a rat model of transient focal cerebral ischemia, melatonin treatment specifically reduced MMP-9 activity without affecting MMP-2 levels [221]. Additionally, melatonin decreased collagen expression in cardiac fibroblasts by inhibiting the NLRP3 inflammasome and the TGF-β1/Smad signaling pathway, thereby alleviating myocardial fibrosis, in diabetic mice [222]. In a unilateral ureteral obstruction (UUO) mouse model of chronic kidney disease, melatonin slowed renal fibrosis by modulating miR-21-5p expression and STAT3 activation, which led to reduced expression of α-SMA, collagen I, and fibronectin [223]. Furthermore, melatonin may reduce soft tissue degradation by decreasing MMP-1 production in periodontal lesions [224]. These modulatory effects of melatonin on MMPs can help prevent abnormal extracellular matrix (ECM) deposition in wound areas.

Most nonhealing wounds do not progress through the normal stages of wound repair but instead remain in a chronic inflammatory state. This persistent inflammation can result in abnormal wound healing, leading to complications such as hypertrophic or keloid scars [225]. Although the precise regulatory mechanism remains unclear, melatonin’s ability to control inflammation in the early stages of wounding may help reduce scar formation during wound healing. Melatonin’s oncostatic potential and its effects on inflammation, collagen production, and fibroblast activity may intersect with the processes involved in hypertrophic scar and keloid formation. Additionally, melatonin possesses potent anti-fibrotic properties, partly through the modulation of MMP activity, and may serve as a valuable therapeutic option for the treatment of fibrosis-related diseases.

### 3.8. Adverse Effect of Melatonin Use

Melatonin is generally considered a safe supplement for short-term use, with a low incidence of serious side effects [18,34,226,227,228,229,230]. However, its bioavailability can be affected by various factors, including interactions with drugs like the CYP1A2 inhibitor fluvoxamine, as well as by the consumption of coffee and vitamins E and C [63]. While melatonin is usually effective, its efficacy can be limited in some cases, and adverse effects, though rare, have been reported. Common side effects include headaches, nausea, and dizziness. Less frequently, melatonin use has been associated with psychiatric issues such as anxiety and depression, skin conditions like rashes and maculopapular eruptions, and gastrointestinal problems including constipation, nausea, and, in rare cases, acute pancreatitis [228,229,230,231,232].

### 3.9. Limitations

In this review paper, we aimed to find the positive roles of melatonin in wound healing and, through this, to investigate the possibility of effective wound management using the evolutionary conserved and versatile molecule melatonin. Wound healing is also complicated and difficult to evaluate in terms of progress, so studies that only evaluate the effect of wound healing are methodologically difficult and have difficulty in interpreting the results. In addition, parameters related to wound healing are often treated as secondary in various clinical settings. However, securing effective and rapid wound healing techniques is important, and the mental and psychological impacts that accompany wounds should not be overlooked.

As the understanding of melatonin’s benefits in wound management continues to evolve, it is important to recognize that our findings may not reflect the latest research. Due to the complexity of wound healing and the limitations in our current knowledge of its mechanisms, studies that focus exclusively on the effects of melatonin in wound healing often encounter methodological challenges and difficulties in result interpretation. Furthermore, research parameters related to wound healing are frequently considered secondary in clinical settings. Nonetheless, the development of effective and rapid wound healing techniques is essential for improving quality of life, as wounds are often accompanied by significant mental and psychological stress.

The precise mechanisms of melatonin’s actions across various clinical contexts require thorough investigation to delineate both receptor-dependent and receptor-independent pathways and to identify specific signaling molecules or pathways that may be targeted by melatonin (Figure 3). The roles of melatonin identified in preclinical and clinical trials should be interpreted with caution until more conclusive evidence is available [33,45,197,233,234]. It is possible that the widely reported benefits of melatonin in different clinical conditions may result from extrapolations due to the use of high doses [33]. Regarding its antioxidant properties, melatonin is suggested to act as a free radical scavenger primarily when administered in pharmacological amounts. However, other studies propose that pharmacological doses of melatonin are necessary to counteract free radical damage, as the production of free radicals in these contexts also occurs at elevated levels [29].

## 4. Conclusions

This review provides a comprehensive overview of scientific studies that highlight melatonin’s potential in promoting effective wound healing (Figure 6). Melatonin, primarily known for its role in regulating sleep and circadian rhythms, also exhibits several properties that may facilitate wound healing and tissue repair. Wound healing is a complex, highly coordinated process involving numerous signaling pathways that work in concert to repair damaged tissues. Melatonin and its metabolites, generated during metabolic processes, demonstrate a broad spectrum of biological activities that support wound healing (Figure 4 and Figure 5), including (1) potent antioxidant effects, (2) anti-inflammatory properties, (3) infection control, (4) regulation of vascular reactivity and angiogenesis, (5) analgesic (pain-relieving) actions, and (6) anti-pruritic (anti-itch) effects. These actions of melatonin may contribute to the promotion and support of wound healing, either independently or in combination with other factors.

Preliminary studies have demonstrated melatonin’s antioxidant, anti-inflammatory, and tissue-regenerative properties, suggesting its potential to promote and support optimal wound healing. However, further research is required to address unresolved questions and determine whether melatonin can be established as a reliable and widely used therapeutic agent in this field. Research on the use of melatonin in promoting wound healing and injury recovery is a rapidly advancing field. Current evidence suggests that melatonin contributes to wound healing through both receptor-mediated and receptor-independent mechanisms. However, the exact pathways by which melatonin enhances this process remain incompletely understood. Some researchers propose that melatonin’s effects may be indirect, improving recovery through better sleep and reduced stress, which are known to influence healing. Others suggest that melatonin has direct effects on cellular processes, such as collagen synthesis and angiogenesis, though these mechanisms require further investigation to confirm their relevance in human wound healing. Therefore, investigating the underlying mechanisms of melatonin’s effects on wound healing at the cellular and molecular levels is necessary. Additionally, our understanding of how melatonin modulates immune responses in wound healing, including its effects on cytokine production, immune cell recruitment, and overall inflammation, should be improved. Numerous pieces of evidence suggest that the efficacy of melatonin in wound healing varies depending on the type and severity of the injury. One of the major controversies surrounds the appropriate dosage and method of administration for melatonin in the context of wound healing. In terms of clinical trials, randomized controlled trials should be conducted to assess the efficacy of melatonin supplementation in various populations, including individuals with chronic wounds, post-surgical patients, and those recovering from traumatic injuries. Exploring different delivery systems for melatonin to enhance its bioavailability and effectiveness is also necessary. These trials could help establish optimal dosages and treatment protocols and, additionally, investigate the synergistic effects of melatonin with other treatments, such as growth factors, stem cell therapy, or other pharmacological agents, to enhance wound healing outcomes.

## Data Availability

Data sharing is not applicable to this article as no datasets were generated or analyzed during the current study.
